# Isolation, Optimization of Fermentation Conditions, and Characterization of an Exopolysaccharide from *Pseudoalteromonas agarivorans* Hao 2018

**DOI:** 10.3390/md17120703

**Published:** 2019-12-13

**Authors:** Lujiang Hao, Wenlin Liu, Kai Liu, Kai Shan, Chunlei Wang, Chenxiang Xi, Jianbang Liu, Qiuping Fan, Xiaofei Zhang, Xiaoping Lu, Yanrui Xu, RuiWen Cao, Yaohong Ma, Lan Zheng, Bo Cui

**Affiliations:** 1 State Key Laboratory of Biobased Material and Green Papermaking, Qilu University of Technology (Shandong Academy of Sciences), Jinan 250353, China; wenlin_liu12@163.com (W.L.); kai_liu2018@163.com (K.L.); kai_shan@163.com (K.S.); chunlei_wang79@163.com (C.W.); ChenXiang_Xi@163.com (C.X.); liujb19960423@163.com (J.L.); fanqiuping1990@163.com (Q.F.); xiaofei_305@163.com (X.Z.); 15269213716@163.com (X.L.); 15269211209@163.com (Y.X.); tiankongxiadebaihe@163.com (R.C.); 2 Kyiv College, Qilu University of Technology, Jinan 250353, China; 3 School of Food Science and Engineering, Qilu University of Technology (Shandong Academy of Sciences), Jinan 250353, China; 4 Biology Institute, Qilu University of Technology (Shandong Academy of Sciences), Jinan 250103, China; mayaohong@126.com (Y.M.); zhlan8409@163.com (L.Z.)

**Keywords:** marine bacteria, fermentation optimization, structural analysis, oxidation resistance

## Abstract

In recent years, the wide application of exopolysaccharides (EPSs) in food, cosmetics, medicine, and other fields has drawn tremendous attention. In this study, an EPS produced by *Pseudoalteromonas agarivorans* Hao 2018 was isolated and purified, and its fermentation conditions were optimized. Its structure and biological functions were also studied. The purity and molecular weight of EPS were determined by high performance liquid chromatography (HPLC), and the EPS exhibited a number average of 2.26 × 10^5^ and a weight average of 2.84 × 10^5^. EPS has good adsorption for Cu^2+^ and Pb^2+^. The adsorption rates can reach up to 69.79% and 82.46%, respectively. The hygroscopic property of EPS was higher than that of chitosan, but slightly lower than that of sodium hyaluronate. However, the water-retaining activity of EPS was similar to that of chitosan and sodium hyaluronate. EPS has strong ability to scavenge free radicals, including OH radical and O^2−^ radical. Further, its activity on O^2−^ radicals has similarities with that of vitamin C. EPS has broad application prospects in many fields, such as cosmetics, environmental protection.

## 1. Introduction

As an important source of life on Earth, the ocean contains abundant biological resources, with prokaryotes accounting for more than 50% of those of the total. Many microorganisms are able to secrete extracellular polymers that are often in the form of exopolysaccharides (EPSs) and are used to protect bacteria from the environmental offenses and for surface adhesion through biofilm formation. The known EPSs of marine bacteria have certain specific characteristics, such as moisture absorption and maintenance, oxidation resistance, and heavy metal ion adsorption. These functions help to compete in their living space to ensure their survival in a complex marine environment and to protect their host from the external adverse environment [1]. In addition, EPSs of marine bacteria have prominent anti-tumor, anti-viral, and immune regulation functions. 

The published research achievements have mainly concentrated on the recent 10 years. Chunlei Wang et al. have successfully isolated and purified the EPSs produced by the marine bacteria *Aerococcus uriaeequi* HZ and explored their biological activity [2]. In this study, the fermentation conditions of marine bacteria *Pseudoalteromonas agarivorans* Hao 2018 were optimized, the optimal fermentation conditions of EPS were explored, and its biological activity was explored. Nadezhda A. Komandrova et al. have reported the structure of *P. agarivorans* KMM232 (R type) [3] and *O*-specific polysaccharides of KMM225T *Pseudoalteromonas* [4], which live in sea ice and seafloor sediments [5], but they also coexist on the surface of some eukaryotes such as sea squirts, sponges, seaweeds, corals, crustaceans, and invertebrate larvae. They often secrete active EPSs [6,7]. Szewzyk U. et al. [8] found that the exopolysaccharide secreted by pseudomonas S9 when it was cultured to a stable period can promote the attachment of S9 on the surface of larvae of the ascidian Ciona intestinalis. When they studied the function of exopolysaccharides during S9 bacteria attaching to the host, it was found that the number of exopolysaccharides secreted by free bacteria or bacteria attached to the surface of the host inorganic substance during nutrient starvation increased. The reduction of the hydrophobicity of the cell surface and the attachment between bacteria and their host can help bacteria escape from a nutrient starvation environment [9,10,11]. Carol Mancuso Nichols et al. [12] studied the effect of temperature on the production of exopolysaccharides from *Pseudomonas* sp. The study also found that CAM025 can produce higher uronic acid under low temperature, and the amount of neutral sugar did not show significant change. Studies on EPS have found that they have many potential biological activities. The high-yield EPS strain *Pseudoalteromonas agarivorans* that Hao 2018, analyzed in this study, was derived from the microbial film of the surface of abalone seedlings. 

In this study, the EPS-producing marine bacteria *Pseudoalteromonas agarivorans* Hao 2018 was screened from the abalone aquaculture base. The EPS produced by the marine bacteria can be fermented in the laboratory [13]. Through the isolation and purification of marine microbial polysaccharides, it is of great importance to screen valuable strains for the production of anti-virus, anti-oxidation, and anti-tumor drugs [14,15,16]. To study EPS, we firstly cultured the strain, and then the extraction, isolation and purification processes were undertaken to prepare EPS. Moreover, we optimized the fermentation conditions. The structure and composition of EPS were analyzed by Fourier transform infrared spectroscopy (FT-IR) and high-performance liquid chromatography (HPLC). Finally, the applications of EPS in modern biotechnology are discussed.

## 2. Results

### 2.1. Extraction and Purification of EPS

**Figure 1:**
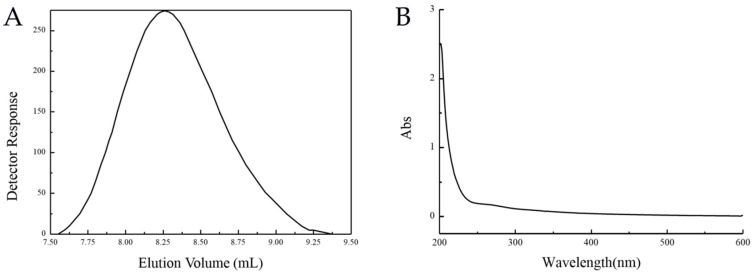
Purification of EPS. The MW distribution of EPS was determined by high performance liquid gel filtration chromatography (HPGFC) using a Shodex SB-806 HQ column with 0.2 M NaCl solution at a flow rate of 0.5 mL/min (**A**). The ultraviolet (UV) visible spectrum of EPS was measured at 200–600 nm in H_2_O at 25 °C (**B**).

*Pseudoalteromonas agarivorans* Hao 2018 was isolated from the Yellow Sea of China. To analyze whether the bacteria can produce EPS, *Pseudoalteromonas agarivorans* Hao 2018 was fermented, and then EPS was isolated using DEAE-52 ion-exchange chromatography and gel filtration chromatography. Yellow-white water-soluble powder was generated through a freeze-drying method. Following high-performance liquid gel filtration chromatography (HPGFC) the purified product produced a single and symmetric peak indicating that EPS was purified in high quality. To examine putative contamination by proteins during the production of EPS, the product needed to be scanned with an ultraviolet visible (UV-Vis) spectrophotometer. EPS did not show absorption at 260 nm and 280 nm in the ultraviolet spectrum, which indicated the absence of nucleic acids and proteins [17]. The total sugar content of EPS was about 2.34 g/L. The molecular weight of EPS was further measured by HPLC, as shown in Figure 1. The average number was 2.26 × 10^5^ g/mol and the average weight was 2.81 × 10^5^ g/mol. The dispersion coefficient was 1.24, indicating that the dispersion level of EPS’s molecular weight was low.

### 2.2. Optimization of Fermentation Conditions

In order to screen for the best fermentation conditions to produce EPS with high efficiency from marine bacteria, the composition of the medium and the fermentation conditions were optimized. Statistical methods such as single factor experimental design, orthogonal experimental design and central combination design were applied to compare the yield of the different conditions. 

#### 2.2.1. Single Factor Experimental Results

**Figure 2:**
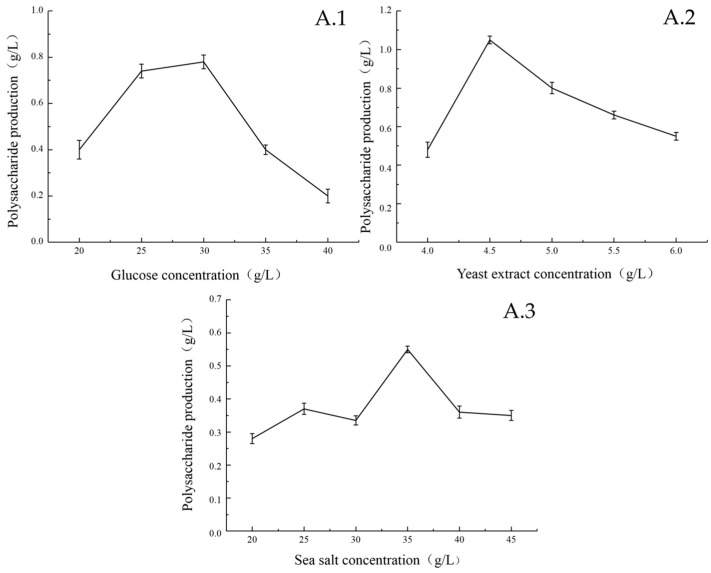
Effects of glucose concentration (**A.1**), yeast extract concentration (**A.2**) and sea salt concentration (**A.3**) on EPS Yield in Medium. Effect of glucose concentration: Sea salt concentration 35 g/L, Yeast extract concentration 4.5 g/L, Temperature 25 °C, Fermentation time 35 h, Seed volume 10%, Shaker speed 230 rpm. Effect of sea salt concentration: glucose concentration 30 g/L, Yeast extract concentration 4.5 g/L, other conditions are the same as above. Effect of yeast extract concentration: glucose concentration 30 g/L, sea salt concentration 35 g/L. Other conditions are the same as above.

The effects of glucose concentration, yeast extract powder concentration and sea salt concentration of different media on the EPS production are shown in Figure 2. When the glucose concentration was 30 g/L, the amount of EPS produced by the strain reached the maximum (Figure 2A.1). This may due to the high concentration of glucose that affects the activity of marine bacteria in the medium, which is detrimental to the EPS production and growth of the strain. When the mass concentration of yeast extract reached 4.5 g/L, the EPS yield of the strain reached the maximum (Figure 2A.2). The strains from the ocean require a certain salinity to grow and produce sugar. Therefore, different concentrations of sea salt were added to the liquid fermentation medium, which greatly influenced EPS production of the strain. The optimal sea salt concentration for EPS production of the strain was 35 g/L (Figure 2A.3). Although this value is high, it is also reasonable because the strain was isolated from marine organisms and is related to its original growth environment.

**Figure 3:**
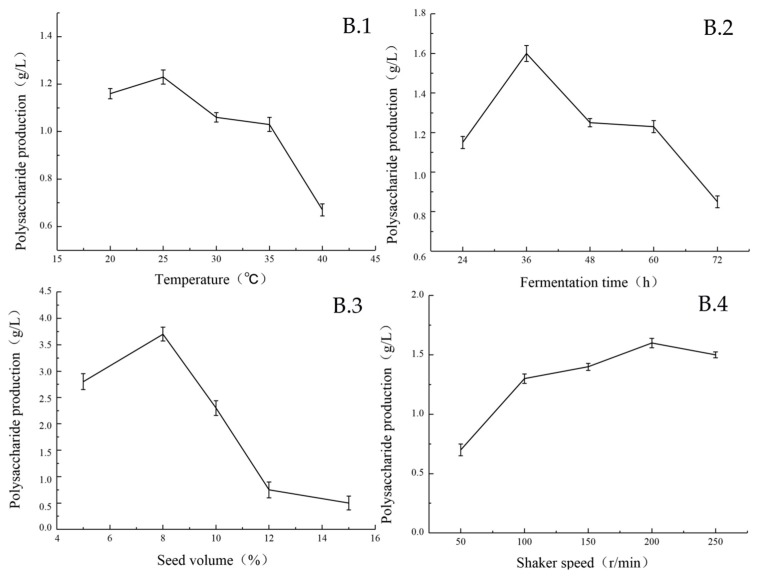
Based on the optimal medium composition of the above experiment (Sea salt concentration 35 g/L, yeast extract concentration 4.5 g/L, glucose concentration 30 g/L), the optimal temperature (**B.1**), fermentation time (**B.2**), seed volume (**B.3**), and shaker speed (**B.4**) are further studied.

The effects of temperature, fermentation time, seed volume and shaker speed on the EPS production by strain under different culture conditions are shown in Figure 3.

**Figure 4:**
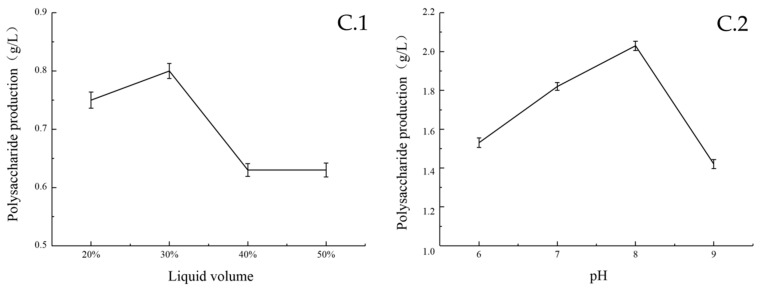
Effect of different liquid volume (**C.1**) and pH (**C.2**) on polysaccharide yield at 25 °C.

Temperature had a great influence on the biomass and sugar yield of the strain. At 25 °C, the biomass and EPS yields reached the maximum level (Figure 3B.1). The content of EPS in the fermentation broth was the highest after 8 h of seed inoculation. The growth curve showed that during this period, bacteria were at the logarithmic phase of growth, with a large number and vigorous vitality. The content of EPS increased steadily within 24–36 h, reached the highest level at 36 h, and then declined (Figure 3B.2). When the seed volume was less than 8%, the yield of EPS was positively correlated with it. When the seed volume was greater than 8%, the yield of EPS was significantly decreased (Figure 3B.3). The reason may be premature consumption of the carbon source by the high number of bacteria introduced by the increased inoculate percentage. The amount of dissolved oxygen in the fermentation process is directly affected by the volume of liquid, which influences the biomass and sugar production of the strain. Among the five horizontal rotational speeds examined in this experiment, the yield of EPS was the highest when the rotational speed of the shaker was 200 rpm (Figure 3B.4). The results are shown in Figure 4. When the liquid volume was 30%, the strain produced the highest amount of EPS (Figure 4C.1). Furthermore, the environmental pH value greatly influenced the biomass and sugar yield of the strain. When the pH value was lower than eight, the EPS yield was at a low level. When the pH was eight, the EPS yield reached the maximum. When the pH was higher than eight, the biomass and EPS yield are tremendously affected by it, and EPS production activity is significantly reduced (Figure 4C.2). 

Therefore, this preliminary experiment determined that the optimal culture temperature was 25 °C, the optimal addition amount was 30%, and the pH value was eight.

#### 2.2.2. Plackett-Burman Results

Significant analysis of experimental factors by Design expert 8.05b, is shown in Table 1, Table 2 and Table 3, and *p* < 0.05 means that the discrepancy is significant. Three factors that had a significant effect on polysaccharide yield are fermentation time, seed volume, and shaker speed. The most important effect on the yield of polysaccharides was the fermentation time, followed by the seed volume. The effect of the shaker speed is relatively small. The concentration of each component in the medium had no significant effect on the yield, so the optimal concentration of each component obtained by the single factor experiment was used as the final composition of the fermentation medium. The composition of the fermentation medium was 30 g/L glucose, 4.5 g/L yeast extract, and 35 g/L sea salt.

#### 2.2.3. Box-Behnken Results

To directly and aptly present the state of the strain under two-factor conditions, the optimal conditions for extracting EPS from bacteria was further evaluated by the response surface method (RSM). The experimental results are shown in Table 4 and Table 5. The *p*-value of this experimental model is much less than 0.01, indicating that this model has a high degree of significance (significant at *p* < 0.05, extremely significant at *p* < 0.001), and the lack of fit *p* = 0.077 < 0.05 indicates that the experimental processes are nearly not affected by other unknown factors. Correlation coefficient *R*^2^ = 0.9885 and correction coefficient *R*^2^_Adj_ = 0.9737 indicate that the regression equation has a good fitting degree and can better simulate the fermentation process of EPS. The *p*-values of the five terms B, A^2^, AB, AC and BC are all less than 0.0001, indicating that inoculation amount, culture time, and shaker speed have a significant effect on the yield of EPS, and the order of the effects is B > A > C.

**Figure 5:**
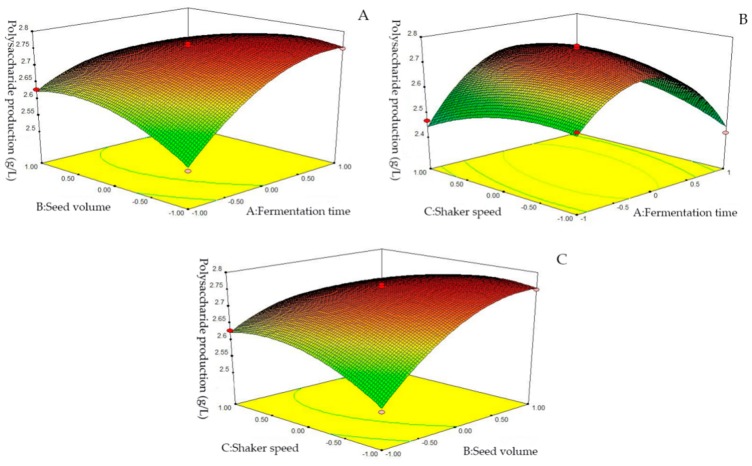
Response surface diagram of two-factor interaction: (**A**) correspondence between the interaction of seed volume and fermentation time; (**B**) correspondence between the interaction of shaker speed and fermentation time; (**C**) correspondence between the interaction of Shaker speed and seed volume.

It is obvious that the fermentation time of the strain had a greater influence on the EPS yield than seed volume and shaker speed. It can be seen from Figure 5C that the seed volume had a greater influence on the EPS yield compared with shaker speed. According to the data analysis, the optimal process conditions for EPS fermentation were as follows: fermentation time 33.78 h, seed volume 8.79%, and shaker speed 171 rpm. Under these conditions, the highest yield of EPS is anticipated to reach up to 2.7836 g/L.

### 2.3. Analysis of Monosaccharide Composition of EPS

**Figure 6:**
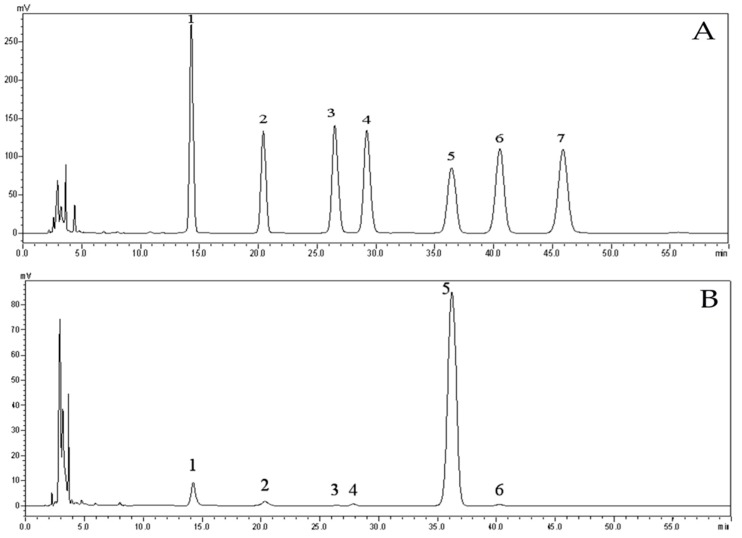
HPLC chromatograms of seven PMP-labeled standard monosaccharides (**A**) and PMP-labeled monosaccharides released from EPS (**B**). Peaks: 1. d-Mannose; 2. d-Rhamnose; 3. d-Glucuronic acid; 4. d-Galacturonic acid; 5. d-Glucose; 6. d-Galactose; 7. *d*-Xylose.

Monosaccharide composition analysis can be adopted to determine the types and quantities of manifold monosaccharides in carbohydrates and glycoproteins. In addition, this information playing a vital role in quantification can be applied to analyze the structure of carbohydrates. EPS was hydrolyzed by sulfuric acid and derivatized with PMP (1-phenyl-3-methyl-5-pyrazolone). Through HPLC, infrared spectroscopy, nuclear magnetic resonance and methylation analysis, six kinds of monosaccharide derivatives such as glucose, mannose, and rhamnose were detected, of which the two main monosaccharides were d-mannose and d-glucose, accounting for 6.66% and 90.28%, respectively (Figure 6).

### 2.4. Fourier Transform Infrared (FT-IR) Analysis

**Figure 7:**
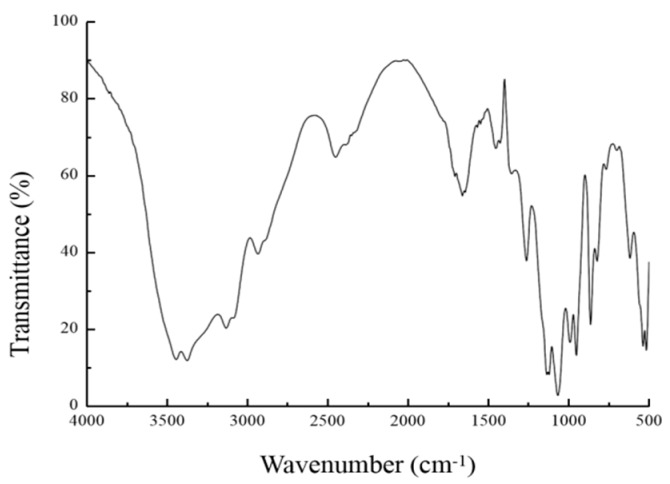
FT-IR spectra of EPS. The dry polysaccharides were crushed and granulated by the KBr method. The ultraviolet-visible spectra of EPS were recorded by a 500–4000 cm spectrophotometer.

FT-IR spectroscopy is a widely used method that represents absorption of infrared light by molecular bonds at a given wavelength [18]. This method can be used to analyze the molecular structure, glycosidic bonds, and functional groups, as well as other polysaccharide structures by studying the vibration of the molecule and the polar bond between the atoms [19,20,21]. As shown in Figure 7, the EPS has an absorption peak peculiar to polysaccharide in the range of 4000 cm^−1^ to 400 cm^−1^, a strong and wide stretching vibration peak in the range of 3500 cm^−1^ and 3300 cm^−1^, which is caused by the intermolecular or intramolecular hydrogen bond formed by –OH on EPS, and a –CH_2_ asymmetric stretching vibration peak of C–H in 2293.73 cm^−1^. There is a stretching vibration peak of C=O at 1682.64 cm^−1^. This characteristic absorption peak may be caused by the acidic polysaccharides. An asymmetric stretching vibration of CO is in 1136.07 cm^−1^, which may be the characteristic absorption peak of pyranose ring. The peak in 864.11 cm^−1^ may be the C–H angular vibration of β-pyranose ring. This characteristic absorption peak confirms the existence of α-d-mannopyranose [22,23]. The results of monosaccharide composition were confirmed by the structure of infrared spectroscopy.

### 2.5. Biological Activity

#### 2.5.1. Adsorption of Cu^2+^ and Pb^2+^ by EPS

**Figure 8:**
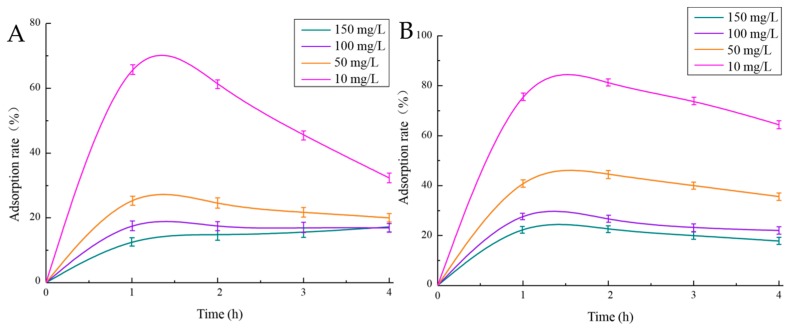
Adsorption rate of EPS for different concentrations of Cu^2+^ (**A**) and Pb^2+^ (**B**).

As shown in Figure 8, when the solution concentration was 10 mg/L and the adsorption time was one hour, the adsorption rates of Cu^2+^ and Pb^2+^ were strongest, reaching 69.79% and 82.46%, respectively. The adsorption effect of EPS on Pb^2+^ was significantly higher than that on Cu^2+^. In addition, the infrared spectra of EPS, EPS + Pb^2+^ and EPS + Cu^2+^ were compared, and it was found that the o-h vibration, C=O stretching vibration and the symmetrical absorption peak of carboxyl group in EPS were shifted after Pb^2+^ and Cu^2+^ were combined, and the chemical structure of EPS changed, which proved the binding of EPS to Pb^2+^ and Cu^2+^ (The equation of standard curve of Cu^2+^ (Figure S1) is: *y* = 0.0088*x* + 0.0331, *R*^2^ = 0.9904, and the degree of fitting was good. Pb^2+^ standard curve is shown in Figure S1, and the linear fitting equation is *y* = 0.0038*x* + 0.0229, *R*^2^ = 0.9907).

#### 2.5.2. Hygroscopicity and Moisture Retention of EPS

**Figure 9:**
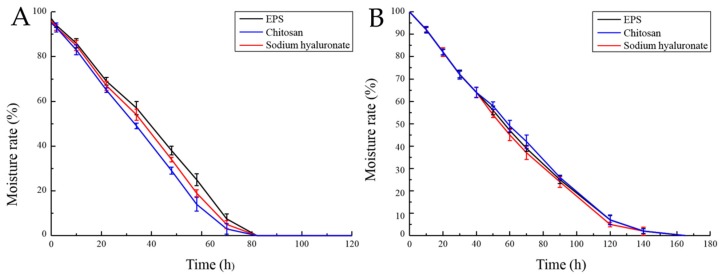
Moisturizing curve under silica gel environment (**A**) and 32% relative humidity (**B**). Moisture retention of EPS was determined by measuring the reserved weight of H_2_O by EPS. Chitosan and sodium hyaluronate were used as controls. The value at the beginning was set 100%.

EPS can maintain a high water content in the microenvironment due to its hygroscopicity [24]. Its hygroscopicity is widely used in food industry [25]. This experiment compared the hygroscopicity of chitosan and sodium hyaluronate with that of EPS. The hygroscopicity of EPS was significantly higher than that of chitosan, but lower than that of sodium hyaluronate (Figure S2). In addition, in cosmetics, the moisture retention of EPS plays an important role [26]. The moisture retention capacity of EPS was studied and compared with that of chitosan and sodium hyaluronate. It is shown that EPS has similar moisture retention capacity with that of chitosan and sodium hyaluronate (Figure 9).

#### 2.5.3. Free Radical Scavenging

**Figure 10:**
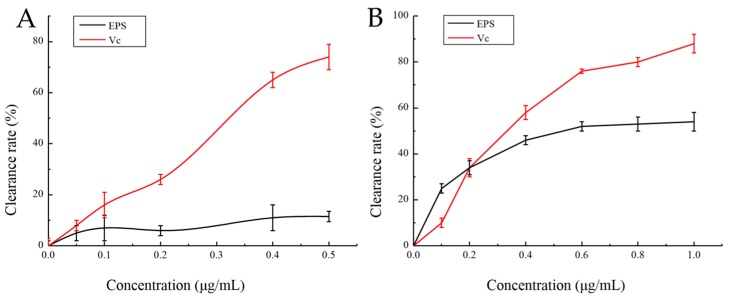
The scavenging effect of EPS on hydroxyl (**A**) and superoxide anions (**B**). The scavenging activities of different concentrations of EPS on –OH and O_2_^−^ were determined by removing –OH from FeSO_4_ and H_2_O_2_ and O_2_^−^ from pyrogallol removal. Vitamin C was used as a control and the activity of 100 µg/mL Vitamin C was set 100%.

Oxygen radicals, such as superoxide radical anions (O_2_^−^) and hydroxyl radicals (–OH), are highly efficient oxidants, which can react with macromolecules in cells and can be mutagenenic and carcinogenic. The possibility of oxidation resistance of polysaccharides from algae, plants, fungi, and prokaryotes as potential therapeutic agents has been studied [27]. For instance, *Dixoniella grisea* (former *Rhodella reticulata*, *Rhodophyta*) EPS has a stronger ability to resist O_2_^−^ The strength of the crude polysaccharide is twice than that of α-tocopherol [28]. EPS from *Brevibacterium* (bacteria, actinomycetes) also has a strong ability to scavenge free radicals, comparable to vitamin C [29]. In this study, the activity of EPS in scavenging –OH·free radicals and superoxide anion O_2_^−^ was analyzed. Figure 10 shows the EPS and Vitamin C (Vc) scavenging curve for hydroxyl free radicals. It can be seen that although the clearance rate of EPS for –OH is far less than that of Vc, it has a certain scavenging effect. In addition, it can also be seen that the scavenging effect of EPS for –OH gradually strengthened with the increase in its concentration, and its highest clearance rate was 12%. Therefore, EPS can be explored as a new potential antioxidant. The activity of EPS in hydroxyl radical scavenging can be attributed to various mechanisms. One possibility is that EPS can absorb free radicals and terminate the free radical reaction.

The clearance curves of EPS and Vc for O_2_^−^ are shown in Figure 10. It can be seen that the scavenging effect of EPS and Vc on O_2_^−^ strengthened with the increase in their concentration. After curve data simulation and correlation analysis, it can be seen that the clearance curve of EPS for O_2_^−^ is in accordance with the logarithmic equation *y* = 18.559Ln(*x*) + 22.914, *R*^2^ = 0.9664, and the correlation is significant. The clearance curve of Vc to O_2_^−^ follows the logarithmic equation *y* = 43.401Ln(*x*) + 8.7824, *R*^2^ = 0.9747, and the correlation is significant. Scavenging rate of superoxide anion reached 50%, also called IC_50_, at 0.43 mg/mL and 0.258 mg/mL concentrations of EPS and Vc, respectively. Although the scavenging effect of EPS on O_2_^−^ is not as good as that of Vc, it is also remarkable.

## 3. Discussion

Microbial EPS has greatly intrigued scientists because of its wide potential. For example, EPS has considerable value in removing pollutants from wastewater, activated sludge dewatering, bioflocculation, and sedimentation. However, there are many factors that influence research and development of EPS. One of the most important factors that restrict its complete commercialization is production cost, especially matrix costs and purification treatment costs [30]. Considering its huge application potential, many studies are being conducted on EPS. Through research on the function of EPS, it has been found that EPS has good hygroscopicity and moisture retention ability and can be on a par with chitosan and sodium hyaluronate, which are commonly used as moisturizers. For example, the study of Sun et al. found that the hygroscopicity of EPS produced by arctic Marine bacteria was higher than chitosan, but lower than hyaluronic acid. Besides, its moisture retention ability was higher than that of chitosan and alginate [31]. However, few studies can clearly explain these properties of EPS. Chen’s research team [32] has reported that the hygroscopicity and moisture retention of carboxymethyl chitosan (chitosan) are related to the active sites of 6-carboxymethyl in the molecular structure. In addition, carboxymethylation at the *N* position promotes moisture absorption and retention, and it increases with molecular weight. According to this study, the main component of EPS is glucose. It can be speculated that the monosaccharide composition of the bacterial EPS and the molecular weight are closely related to its hygroscopicity and moisture retention. The differences in the mechanism between hygroscopicity and moisture retention need to be revealed. It is important to note that biomaterials with hygroscopicity and moisture retention abilities have been widely applied to cosmetics, food, pharmacy and other industries [33]. EPS has great potential to become wound dressing and moisturizing ingredient because of its moisture retention ability [34]. Furthermore, the oxidation resistance of EPS is important to maintain human health and prevent disease.

## 4. Materials and Methods

### 4.1. Separation and Purification of EPS

The strain selected in this study was isolated from the microbial membrane on the surface of abalone seedlings and had the ability to produce exopolysaccharides. The marine bacterium *Pseudoalteromonas agarivorans* Hao 2018 was cultured for 8 h in an Erlenmeyer flask at 25 °C. The seed broth was Zobell 2216E liquid medium, and its composition was: peptone 5 g/L, yeast extract 1 g/L, sea salt 35 g/L, pH 8. 10% inoculate was added into fermentation medium and cultured at 25 °C for 35 h while shaking at 230 rpm. The fermentation medium composition was glucose 30 g/L, yeast extract 4.5 g/L, and sea salt 35 g/L. The fermentation broth was centrifuged at 6000 rpm for 5 min. The supernatant was collected, 4 volumes of 95% ethanol were added and incubated overnight at 4 °C to precipitate [35]. Then, the mixture was centrifuged at 6000 rpm for 5 min. The supernatant was removed, and the precipitate was poured into water and mixed with savage reagent (chloroform: *n*-butanol = 5:1) at a volume ratio of 1:4. The solution was mixed thoroughly and centrifuged to remove the organic solvent and denatured proteins [36]. To determine the total sugar content of EPS, the phenol-sulfuric acid method was adopted [37]. In this experiment, polysaccharides were first hydrolyzed into monosaccharides under the function of sulfuric acid, and quickly dehydrated to form sugar-aldehyde derivatives, and then formed orange-yellow compounds with phenol, and then determined by colorimetric method. The EPS in deionized water was further purified with 1.6 × 30 cm column through DEAE-52 anion exchange chromatography. The samples were eluted with a linear gradient of 0~1 M NaCl solution at a flow rate of 60 mL/h. The EPS was further purified in a column (1.6 × 100 cm) through gel filtration chromatography and eluted with 0.1 mL NaCl solution [38] at a flow rate of 12 mL/h. Then, the purified EPS was dialyzed with deionized water and a selective semi-permeable membrane (8000~14,400 da).

### 4.2. Optimization of Fermentation Conditions

#### 4.2.1. Single Factor Experiment Method

Factors that may affect EPS production during fermentation include the carbon source, nitrogen source, and sea salt of fermentation medium, as well as temperature, pH value, liquid volume, seed age, inoculum amount, and shaker speed. The impact of these factors was examined in single factor experiments as follows:

Different kinds of carbon and nitrogen sources were examined for the fermentation of EPS. After obtaining the best carbon and nitrogen sources, the experiment was carried out under different concentrations in order to determine the most suitable concentration.

Regarding sea salt and other fermentation factors, single factor experiments were performed with different levels of each factor to determine the optimal levels.

#### 4.2.2. Plackett-Burman Design

According to the results of the single factor experiments, the factors that greatly affected EPS yield were selected. The importance of nine factors including temperature, liquid volume, culture time, pH value, inoculation amount, shaker speed, as well as carbon source, nitrogen source and sea salt of the culture medium were investigated. Each factor had two levels, high and low, to screen for significant factors influencing EPS production. The specific design method is shown in Table 6.

#### 4.2.3. Box-Behnken Design

According to the significant factors determined by the Plackett-Burman design, the Box-Behnken design was applied to select three levels of each factor. The experimental data were processed by Design-Expert 8.05b software in order to obtain multivariate fitting equations and response surface graphs. The specific design method is shown in Table 7.

#### 4.2.4. Data Statistical Analysis

The results of single factor experiments were analyzed by Excel spreadsheet, and the data collected by Plackett-Burman and Box-Behnken design were processed and analyzed by Design-Expert software to determine the most significant factor affecting EPS yield, and to obtain the multivariate fitting equation and response surface graphs of polysaccharide yield.

### 4.3. Determination of Polysaccharide Content

In this experiment, the phenol-sulfuric acid method was used to determine the polysaccharide yield of *Pseudoalteromonas agarivorans* Hao 2018 fermentation broth [37]. At first, the glucose standard curve was drawn: 20 mg glucose was dissolved to 500 mL water, and water was added to aliquots of 0, 0.4, 0.6, 0.8, 1.0, 1.2, 1.4, 1.6, and 1.8 mL glucose solution up to 2.0 mL. Then, 1 mL 6% phenol and 5 mL concentrated sulfuric acid were added. The reaction solution was allowed to stand for 10 min, then shaken, and reacted at 25 °C for 20 min. The absorbance of the reaction solution was determined at 490 nm. As blank control 2.0 mL distilled water was used. The standard curve was drawn with glucose mass as the horizontal coordinate and absorbance as the vertical coordinate. The fermentation broth of *Pseudoalteromonas agarivorans* Hao 2018 was centrifuged (6000 rpm, 5 min) to remove the cells, and the supernatant was collected, and four times the volume of 95% ethanol was added. The solution was completely shaken and allowed to stand at 4 °C overnight. Next, the precipitate was collected by centrifugation (6000 rpm, 5 min) and diluted with water to a suitable concentration. An aliquot of 1 mL was used in the experimental procedure. The EPS content was calculated using the glucose standard curve.

### 4.4. Determination of the Molecular Weight of EPS

To determine the molecular weight of EPS, gel filtration chromatography was used [39]. HPGFC with a Shodex SB-806 HQ column (0.8 × 30 cm) was used. 0.2 M NaCl in H_2_O was included in mobile phase. The system was run at 35 °C at a flow rate of 0.5 mL/min. Then, 100 μL of the standard sample (Table 8) or the EPS sample were added into the liquid chromatograph. Then the chromatograms were recorded. GPC software was used to correct the linear regression equation. The standard curve is shown in Figure S3. The reference material had a k value of 0.0006 and an α value of 0.75.

#### 4.4.1. Hydrolysis and Derivatization of EPS

Twenty mg EPS were mixed with 10 mL sulfuric acid solution (1 M/L) and incubated at 100 °C for 8 h in order to hydrolyze it. After that, the hydrolyzate was centrifuged at 10,000 rpm for 10 min. An aliquot of 4.5 mL of the supernatant was taken, and 2 mol/L NaOH was used to neutralize the pH of the supernatant. Finally, the neutralization solution was added to attain final volume of 10 mL. The PMP derivatization of monosaccharides was suitably modified as previously described [40,41]. Briefly, 50 μL neutralized solution, 50 μL PMP-methanol solution (0.5 mol/L) and 50 μL sodium hydroxide solution (0.3 mol/L) were mixed together in a 1.5 mL tube and placed in a water bath at 70 °C for 30 min. After it cooled to 25 °C, 50 μL of HCl solution were added to the mixture to neutralize the pH and 100 μL of ultrapure water to dilute it. In addition, the mixture was extracted with chloroform three times, and filtered through a 0.45-micron filter before being applied to high performance liquid chromatography (HPLC). At the same time, the standard solutions of 2 mM xylose, arabinose, glucopyranose, mannose, and fucose were processed in the same way. All tests were conducted independently three times.

#### 4.4.2. Analysis of Monosaccharide Composition by High Performance Liquid Chromatography (HPLC)

HPLC, which consists of a mobile phase of 80% ammonium acetate solution and 20% acetonitrile at a flow rate of 1.0 mL/min at 30 °C was equipped with an InertSustain (4.6 × 250 mm) column and used to identify and quantify monosaccharide composition of EPS. EPS was detected at 245 nm using a detector [42].

#### 4.4.3. Ultraviolet (UV) Visible Light and FT-IR Spectrum

The UV visible spectrum of EPS was recorded by a UV-2450 spectrophotometer (Shimadzu, Kyoto, Japan). FT-IR (PerkinElmer, Norwalk, CT, USA) was used to record the FT-IR spectrum of the sample [43].

### 4.5. Biological Activity

#### 4.5.1. Adsorption of Cu ^2+^ and Pb ^2+^ by EPS

A solution of 2 g/L CuSO_4_ was prepared for use in a 10 mL volumetric flask and diluted to 10, 50, 100, and 150 mg/L, respectively. The Cu^2+^ content in the solution was determined by atomic absorption spectrophotometer (flame method).

A solution of 1 g/L EPS polysaccharide was prepared in a 100 mL volumetric flask and diluted to 50 mg/L. Forty mL of EPS solution were poured it into a selective semi-permeable membrane (retained molecular weight 8000–14400), and the bottle mouth tightene. Two hundred mL of 10, 50, 100, 150 mg/L CuSO_4_ solution were added to each 500 mL flask. The dialysis bag was completely immersed in the solution contained within each flask and incubated at 4 h at 25 °C while stirring at 200 rpm. An aliquot was withdrawn every hour, and the content of Cu^2+^ in the reaction solution was measured by the atomic absorption method. The corresponding Cu^2+^ clearance rate was calculated according to the Cu^2+^ standard curve, and a curve was drawn.

To measure the concentration of Pb^2+^ the same method as above was used but the CuSO_4_ solution was changed to Pb(NO_3_)_2_. The clearance rate of Pb^2+^ by the EPS solution was calculated, and the corresponding curve was drawn.

#### 4.5.2. EPS Hygroscopic Activity

Flasks containing 0.1 g EPS were placed in two sealed desiccators with saturated NaCl or CaCl_2_ solution whose relative humidity was 73% and 32%. After the set time, the sample was weighed, and the moisture absorption rate was calculated. Chitosan (Hyderabad Marine Biological Engineering Co., Ltd., FG) and sodium hyaluronate (Furida Biomedical Co., Ltd., external use) were used as controls. The calculation of hygroscopic rate was based on the equation:

Hygroscopic Rate (%) = (increased weight/original weight) × 100%

#### 4.5.3. Determination of Moisture Retention Rate of EPS

First, 0.1 g EPS in 0.5 g distilled water in a flask were incubated in the sealed dryers with a saturated CaCl_2_ solution or silica gel, respectively. The relative humidity in the two dryers was 32% and 0%. After the set time, the sample was weighed, and the moisture absorption rate was calculated. The results were compared with that of chitosan and sodium hyaluronate. The calculation of moisture retention is based on the equation:

Moisture Retention (%) = (weight of water after incubation/original weight of water) × 100%

#### 4.5.4. OH Free Radical Scavenging Activity

The Fenton reaction was used to analyze the free radical scavenging activity of EPS for –OH [44]. Hydroxyl radicals, 0.5 mL FeSO_4_ (7.5 mM) and 0.5 mL H_2_O_2_ (3%, *v*/*v*) were mixed with 1.0 mL 1,10-phenanthroline (5 mM) and 1.0 mL sodium phosphate buffer solution (0.05 M, pH 7.4). A series of different concentrations of EPS (0–0.5 mg/mL) were added to the solution and then the mixture was allowed to react at 37 °C for 30 min. The absorbance was measured at 510 nm. H_2_O and Vc were used as a blank control and positive control, respectively. The calculation formula of clearance was:

Clearance Rate (%) = [A_0_ − (A_x_ − A_x0_)]/A_0_ × 100%

In the above formula, ΔA_0_ and ΔA represent the absorbance of blank solution and that of the solution with EPS added, respectively. A_x0_ symbolizes the absorption of the polysaccharide solution background.

#### 4.5.5. O^2−^ Free Radical Scavenging Activity

To determine the scavenging activity of O^2−^ by EPS in vitro, the pyrogallol autooxidation method was used. In short, the following reagents were added into a cuvette in the order: 10 μL pyrogallol (3 mM), 80 μL NaOH (4 mM), 10 μL EPS and 900 μL luminol (0.1 mM in sodium carbonate buffer solution, pH = 10.2) and then incubated in water at 25 °C. Finally, a series of reactions with the different concentrations of EPS were performed, and the absorbance was recorded at 325 nm. The vitamin C group served as a positive control. The calculation formula was:

Clearance Rate (%) = (∆A_0_ − ∆A)/(∆A_0_) × 100%

where ΔA_0_ and ΔA represent the autoxidation rate with EPS and that without EPS, respectively.

## 5. Conclusions

In this study, the fermentation conditions of *Pseudoalteromonas agarivorans* Hao 2018 strain were optimized and the optimal fermentation conditions were established. The optimal formula of fermentation medium includes 30 g/L glucose, 4.5 g/L yeast extract, 35 g/L sea salt, and the optimal fermentation conditions are as follows: 33.8 h culture time, 8.8% inoculation, 170 rpm shaker speed. Under all these conditions, the average yield of EPS can reach 2.604 ± 0.022 g/L. The EPS was obtained by purification and the purity and molecular weight of EPS were determined by HPGFC, and the results were *M*_n_ = 2.26 × 10^5^ g/mol, *M*_w_ = 2.81 × 10^5^ g/mol. The dispersion coefficient was 1.24, and the degree of dispersion was small. The relative purity of EPS was uniform. The study found that the main component of EPS is glucose, accounting for more than 90.28%. In addition, it was found that EPS has good hygroscopicity and moisture retention ability, and can be on a par with chitosan and sodium hyaluronate, which are commonly used as moisturizers. EPS displayed a certain scavenging ability for OH, and the highest clearance rate was 12%. Furthermore, the scavenging effect of EPS on O_2_^−^ was significant. EPS had good adsorption for Cu^2+^ and Pb^2+^. The adsorption rates can reach up to 69.79% and 82.46%, separately, and the adsorption effect of EPS on Pb^2+^ was significantly higher than that of Cu^2+^.

## Figures and Tables

**Table 1:** Plackett–Burman experimental design results.

Serial Number	A	B	C	D	E	F	G	H	J	K	L	Polysaccharide Production (g/L)
1	1	1	−1	−1	−1	1	−1	1	1	−1	1	2.315
2	1	−1	1	1	1	−1	−1	−1	1	−1	1	2.629
3	−1	1	1	−1	1	1	1	−1	−1	−1	1	2.573
4	−1	−1	−1	1	−1	1	1	−1	1	1	1	2.444
5	−1	−1	1	−1	1	1	−1	1	1	1	−1	2.621
6	1	1	−1	1	1	1	−1	−1	−1	1	−1	2.694
7	−1	−1	−1	−1	−1	−1	−1	−1	−1	−1	−1	2.653
8	1	−1	1	1	−1	1	1	1	−1	−1	−1	2.726
9	−1	1	−1	1	1	−1	1	1	1	−1	−1	2.637
10	1	1	1	−1	−1	−1	1	−1	1	1	−1	2.879
11	−1	1	1	1	−1	−1	−1	1	−1	1	1	2.935
12	1	−1	−1	−1	1	−1	1	1	−1	1	1	3.024

**Table 2:** Significant analysis.

Factor	Regression Coefficients	Impact Level	Contribution
A	0.034	0.067	3.13
B	−5.333 × 10^−003^	−0.011	0.079
C	0.05	0.099	6.82
D	0	0	0
E	0.019	0.0038	0.98
F	−0.12	−0.23	36.76
G	0.036	0.073	3.65
H	0.032	0.064	2.86
J	−0.09	−0.18	22.38
K	0.089	0.18	21.73
L	−0.024	−0.048	1.61

**Table 3:** Significant analysis results.

Source of Variation	Sum of Squares	Degree of Freedom	Mean Square	*F* Value	*P* Value
Model	0.35	3	0.12	11.27	0.003
Fermentation time	0.16	1	0.16	15.37	0.0044
Seed volume	0.097	1	0.097	9.36	0.0156
Shaker speed	0.094	1	0.094	9.09	0.0167

**Table 4:** Box-Behnken experimental design results.

Serial Number	A	B	C	Polysaccharide Production (g/L)
1	−1	1	0	2.637
2	0	0	0	2.766
3	0	0	0	2.758
4	−1	−1	0	2.298
5	−1	0	−1	2.573
6	0	−1	1	2.629
7	0	0	0	2.742
8	1	0	1	2.548
9	0	−1	−1	2.508
10	0	0	0	2.766
11	1	0	−1	2.419
12	1	−1	0	2.556
13	1	1	0	2.460
14	0	1	−1	2.750
15	−1	0	1	2.468
16	0	0	0	2.742
17	0	1	1	2.661

**Table 5:** Box-Behnken experimental analysis of variance table.

Source of Variation	Sum of Squares	Degree of Freedom	Mean Square	*F* Value	*p* Value
Model	0.32	9	0.036	66.81	<0.0001
A-Fermentatio*n* time	6.125 × 10^−6^	1	6.125 × 10^−6^	0.012	0.9175
B-Seed volume	0.033	1	0.033	62.87	<0.0001
C-Shaker speed	3.920 × 10^−4^	1	3.920 × 10^−4^	0.74	0.4189
AB	0.047	1	0.047	89.02	<0.0001
AC	0.014	1	0.014	25.76	0.0014
BC	0.011	1	0.011	20.75	0.0026
A^2^	0.17	1	0.17	320.17	<0.0001
B^2^	0.018	1	0.018	34.54	0.0006
C^2^	0.011	1	00.011	21.24	0.0025
residual	4.1184 × 10^−3^	9	4.576 × 10^−4^		
Lack of fit	3.529 × 10^−3^	5	7.059 × 10^−4^	4.80	0.077
Net Errors	5.888 × 10^−4^	4	1.472 × 10^−4^		
Total deviation	0.32	16			

**Table 6:** Factors and levels of Plackett-Burman design.

Variable	Factor	Low Level (−)	High Level (+)
A	Glucose content	30 g/L	35 g/L
B	Yeast extract content	2.5 g/L	5 g/L
C	Sea salt content	30 g/L	35 g/L
E	Liquid volume	30%	40%
F	Cultivation time	36	48
G	pH	7	8
J	Seed volume	8%	10%
K	Shaker speed	100 rpm	200 rpm
L	Temperature (°C)	25	30

**Table 7:** Factors and levels of Box-Behnken design.

Factor	Number	Level		
		−1	0	1
Culture time	A	30 h	36 h	42 h
Seed volume	B	7%	8%	9%
Shaker speed	C	150 rpm	200 rpm	250 rpm

**Table 8:** Standard samples of the PSS.

Standard Samples LOT	MW (g/mol)	Weight (mg)
PSS929n	63,900	10.0
PSS8065n	152,000	10.2
PSS13092	282,000	10.1
PSS14052	976,000	10.0
PSS9304-4	2,260,000	10.1

## Supplementary Materials

The following are available online at https://www.mdpi.com/1660-3397/17/12/703/s1, Figure S1: Standard curve equation of Cu^2+^ and Pb^2+^; Figure S2: Hygroscopicity of chitosan, sodium hyaluronate and EPS; Figure S3: Standard curve of HPGFC series of standard samples.
