# Supplementary material for: Isolation, Optimization of Fermentation Conditions, and Characterization of an Exopolysaccharide from Pseudoalteromonas agarivorans Hao 2018
Source: Mar Drugs. 2019 Dec 13;17(12):703. doi: 10.3390/md17120703 (PMC6950073; doi:10.3390/md17120703)
Supplement: Supplementary file 1 [file marinedrugs-17-00703-s001.pdf]

marine drugs

MDPI

Supplementary Materials

Isolation, Optimization of Fermentation Conditions, and Characterization of an Exopolysaccharide from *Pseudoalteromonas agarivorans* Hao 2018

Lujiang Hao [1,2*†], Wenlin Liu [1†], Kai Liu [1], Kai Shan [1], Chunlei Wang [1], Chenxiang Xi [1], Jianbang Liu [1], Qiuping Fan [1], Xiaofei Zhang [1], Xiaoping Lu [1], Yanrui Xu [1], RuiWen Cao [1], Yaohong Ma [4], Lan Zheng [4], Bo Cui [3*]

[1] State Key Laboratory of Biobased Material and Green Papermaking, Qilu University of Technology (Shandong Academy of Sciences), Jinan 250353, China;wenlin_liu12@163.com(W.L.); kai_liu2018@163.com(K.L.); kai_shan@163.com(K.S.); chunlei_wang79@163.com(C.W.); ChenXiang_Xi@163.com(C.X.); liujb19960423@163.com(J.L.); fanqiuping1990@163.com (Q.F.); xiaofei_305@163.com (X.Z.); 15269213716@163.com (X.L.); 15269211209@163.com (Y.X.); tiankongxiadebaihe@163.com(R.C.)

[2] Kyiv College at Qilu University of Technology, Jinan 250353, China

[3] School of Food Science and Engineering, Qilu University of Technology (Shandong Academy of Sciences), Jinan 250353, China

[4] Biology Institute, Qilu University of Technology (Shandong Academy of Sciences), Jinan 250103, China;mayaohong@126.com(Y.M.); zhlan8409@163.com(L.Z.)

* Corresponding Author. Email: lujiang_hao@qlu.edu.cn; cuibopaper@163.com

† These authors contributed equally to this work

Figure S1

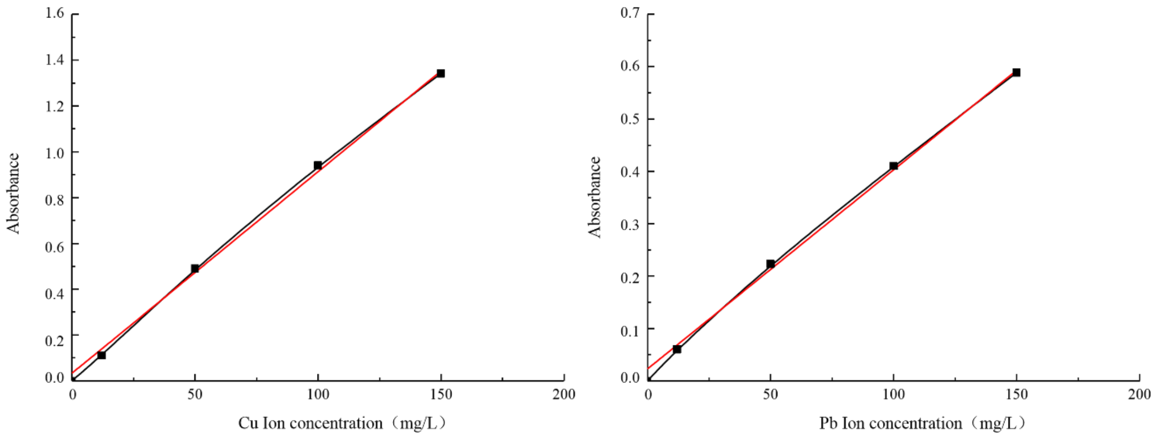

Figure S1. The equation of standard curve of Cu^{2+} is: y=0.0088x+0.0331, R2=0.9904, and the degree of fitting was good. The equation of standard curve of Pb^{2+} is: y=0.0038x+0.0229, R2=0.9907

Figure S2

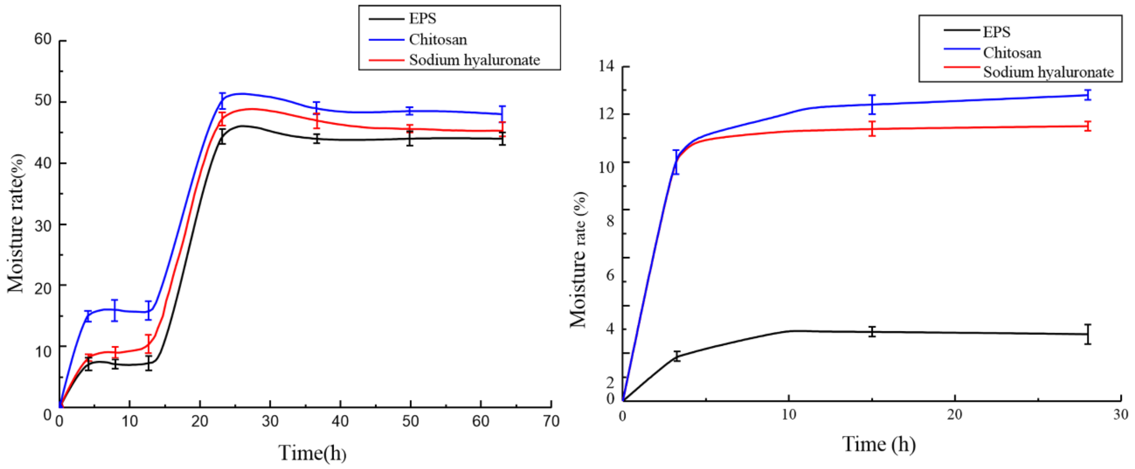

Figure S2. The hygroscopicity of chitosan, sodium hyaluronate and EPS was compared by experiments. The hygroscopicity of EPS is significantly higher than that of chitosan, but lower than that of sodium hyaluronate.

Figure S3

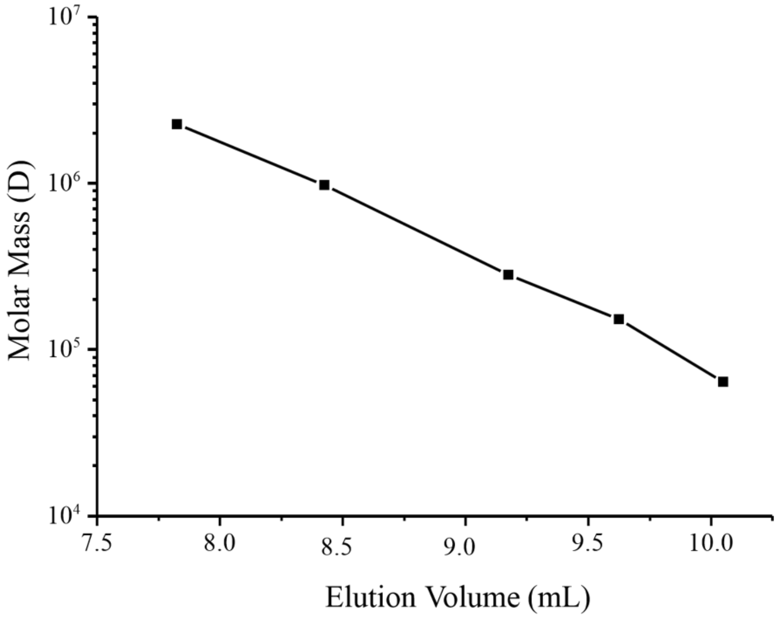

Figure S3. 100 μL standard sample or EPS sample was added to the liquid chromatograph. Then record the chromatogram. The linear regression equation was corrected using GPC software to obtain a standard curve.
